# P367: Airborne decontamination system in hospital background

**DOI:** 10.1186/2047-2994-2-S1-P367

**Published:** 2013-06-20

**Authors:** S Loeffert, A Hautemaniere

**Affiliations:** 1 Meurthe et Moselle, University of Lorraine, Nancy, France

## Introduction

Since the commercial use of Formalin chemicals, classified as carcinogenic, has been forbidden, Airborne Decontamination Systems (ADS) have been revalued by the use of new disinfection products and technology.

## Objectives

The aim of this review was to review all ADS of environmental surfaces which have been tested until today to control the spread of hospital-acquired pathogens and infections.

## Methods

Research were conducted online using PubMed. All reports with experimental study about the impact of ADS on nosocomial pathogens were included. The main key words used for this selection were: air disinfection & hydrogen peroxide, aerosol & disinfection & efficacy, aerosol & hydrogen peroxide vapour. The selected papers included in the review were those in which we could calculate the logarithm reduction due to ADS. A comparison was made according to the products used, the different systems applied and the efficacy found.

## Results

In the end, 21 papers consisting of 28 ADS experiments were included in this review. A large variety of products used for ADS are presented i.e.: hydrogen peroxide (H_2_O_2_), ozone (O_3_), metastable hypochlorus acid (HA), solution of bioflavanoids & fruit acids extracts (Citrox), triethylene glycol (TEG) and gas plasma. 18 papers have studied ADS using H_2_O_2_ with a range of concentration from 12 ppm to 700 ppm. Three papers used ADS with a range of O_3_ going from 0.04 ppm to 25 ppm. One paper presented ADS using a concentration of about 2 ppm of TEG. Another study compared ADS system with 1000 ppm of metastable HA and ADS with a 5% Citrox solution. A part of human pathogens frequently found in hospitals and more precisely in patient surroundings were exposed to this variety of ADS. According to each study, location, exposure time and concentration of inoculums differ. In a general way, after ADS exposition the logarithm reduction observed is comprised between 0.33- log_10_ and over 7-log_10_. The ADS which seems to be the most effective against a large range of germs is the H_2_O_2_ vapor systems with a logarithm reduction ranged from <0.6-log_10_ to >7-log_10_.

## Conclusion

Despite all the new technology and products created for ADS and the lack of international standard, this review suggests that their efficacy is depending on the products used, the experimental conditions applied and the germs tested.

## Disclosure of interest

None declared

